# Relação entre o Escore Prognóstico de Nápoles e a Doença do Enxerto de Veia Safena após Cirurgia de Revascularização do Miocárdio

**DOI:** 10.36660/abc.20240519

**Published:** 2025-05-15

**Authors:** Ahmet Karaduman, Cemalettin Yılmaz, Muhammet Mucahit Tiryaki, İsmail Balaban, Mustafa Ferhat Keten, Tuba Unkun, Servet İzci, Suleyman Çağan Efe, Elnur Alizade

**Affiliations:** 1 Department of Cardiology Kartal Kosuyolu Research and Education Hospital Istanbul Turquia Department of Cardiology, Kartal Kosuyolu Research and Education Hospital, Istanbul – Turquia; 2 Department of Cardiology Mus State Hospital Mus Turquia Department of Cardiology, Mus State Hospital, Mus – Turquia

**Keywords:** Revascularização Miocárdica, Veia Safena, Inflamação, Aterosclerose

## Abstract

**Fundamento:**

A permeabilidade do enxerto de veia safena (EVS) continua sendo um desafio em casos de doença arterial coronária após cirurgia de revascularização do miocárdio (CRM). O escore prognóstico de Nápoles (NPS) constitui um novo sistema de pontuação projetado para avaliar tanto o estado nutricional quanto a inflamação.

**Objetivos:**

Nosso estudo teve como objetivo explorar a associação entre NPS e doença do EVS em pacientes com histórico prévio de CRM.

**Métodos:**

Foram revisados um total de 702 pacientes submetidos à CRM e à angiografia coronáriaretrospectivamente. A doença do EVS foi definida como a presença de estenose ≥50% em pelo menos um EVS. Os pacientes foram categorizados em dois grupos com base na presença ou ausência de doença do EVS. Valores de p<0,05 foram aceitos como estatisticamente significativos.

**Resultados:**

A população do estudo consistiu em 702 pacientes, com 269 (38,3%) apresentando EVSs degenerativos e 433 (61,7%) sem EVSs degenerativos. O NPS foi maior no grupo com degeneração da veia safena e surgiu como um preditor significativo de doença do EVS (OR: 1,596, IC 95%: 1,198-2,125, p=0,001). Além disso, hipertensão (OR: 2,344, IC 95%: 1,137-4,833, p=0,02), doença renal crônica (OR: 3,337, IC 95%: 1,554-7,168, p=0,002), uso de estatina (OR: 0,434, IC 95%: 0,239-0,789, p=0,006), intervalo de tempo desde a CRM (OR: 1,138, IC 95%: 1,213-1,432, p<0,001) e número de EVSs (OR: 2,708, IC 95%: 1,902-3,855, p<0,001) foram preditores significativos da doença do EVS.

**Conclusão:**

O NPS, uma ferramenta útil para avaliar inflamação e estado nutricional, pode fornecer informações valiosas sobre a permeabilidade de EVSs após cirurgia de CRM. Pacientes com NPS elevado após CRM devem passar por monitoramento cuidadoso para o desenvolvimento de doença de EVS.

## Graphical abstract

**Figura Central:**
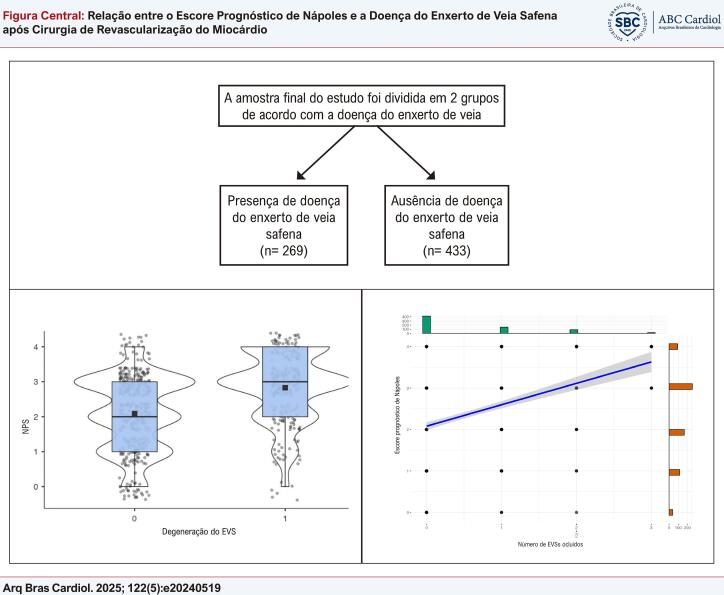
Relação entre o Escore Prognóstico de Nápoles e a Doença do Enxerto de Veia Safena após Cirurgia de Revascularização do Miocárdio

## Introdução

A cirurgia de revascularização do miocárdio (CRM) é uma abordagem terapêutica eficaz utilizada por muitos anos para aliviar episódios de angina, melhorar a qualidade de vida e estender a expectativa de vida de pacientes com doença arterial coronária. Tanto os enxertos arteriais quanto os venosos são opções viáveis para esse procedimento. No entanto, em comparação aos enxertos arteriais, os enxertos de veia safena (EVSs) têm taxas de permeabilidade mais baixas, particularmente em contraste com a artéria mamária interna. Aproximadamente 12% dos EVSs sofrem oclusão dentro de um mês após a cirurgia, com a taxa de oclusão aumentando para 40% no décimo ano após a cirurgia de CRM.^1,2^ Vários mecanismos foram postulados para a doença do EVS, incluindo trombose, hiperplasia intimal e aterosclerose.^3,4^ Além dos fatores de risco de aterosclerose estabelecidos, fatores como a idade do EVS e o diâmetro da veia nativa em procedimentos de CRM também podem contribuir para o desenvolvimento de patologias de EVS.^5^ A importância de cada mecanismo pode variar entre os pacientes, estimulando esforços de pesquisa com o objetivo de identificar indicadores de risco para a doença do EVS.

O escore prognóstico de Nápoles (NPS) constitui um sistema de pontuação recentemente desenvolvido que incorpora níveis de albumina sérica e colesterol total, juntamente com a razão neutrófilo-linfócito (RNL) e razão linfócito-monócito (RLM), visando avaliar o estado nutricional e inflamatório dos pacientes.^6,7^ Inicialmente validado como um indicador prognóstico para pacientes submetidos à cirurgia de câncer colorretal, o NPS foi mais recentemente associado a resultados adversos em indivíduos com síndrome coronariana aguda e insuficiência cardíaca (IC).^8-10^ Apesar do estabelecimento bem-sucedido de associações entre prognóstico do paciente e NPS em várias coortes de doenças cardiovasculares, há uma notável falta de pesquisas abordando sua relevância para a permeabilidade do EVS. Portanto, nosso estudo teve como objetivo explorar a relação entre NPS e doença do EVS em pacientes com histórico de cirurgia de CRM.

## Métodos

### População do estudo

Este estudo observacional retrospectivo incluiu pacientes que haviam passado por CRM e posteriormente passaram por angiografia coronária entre janeiro de 2016 e maio de 2024. Todos os pacientes apresentaram sintomas de angina estável e/ou resultados positivos de teste de estresse ou síndrome coronária aguda. Dados clínicos, demográficos e laboratoriais foram coletados por meio de uma revisão de prontuários médicos. Informações sobre os medicamentos usados pelos pacientes antes do procedimento de angiografia coronária também foram documentadas. Os resultados basais da angiografia coronária foram analisados e o intervalo de tempo médio entre o CRM e a angiografia coronária basal foi calculado.

**Figura 1:**
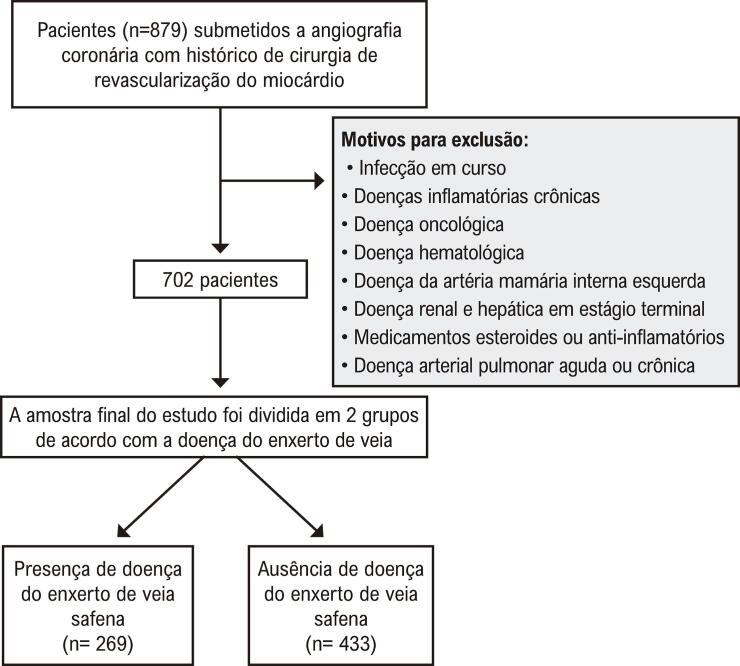
– CONSORT da população do estudo.

Os critérios de exclusão incluíram doença cardíaca valvular significativa, IC descompensada, doença pulmonar aguda ou crônica, doença autoimune, doença infecciosa ou inflamatória, doença da artéria mamária interna esquerda, uso de esteroides ou medicamentos antiinflamatórios e histórico dedistúrbios hematológicos ou malignidade. Após a triagem para os critérios de inclusão e exclusão, 702 pacientes com EVS foram incluídos na análise final. O fluxograma da população do estudo é mostrado na Figura 1. Esses pacientes foram categorizados em dois grupos com base na permeabilidade do EVS.

Hipertensão (HT) foi definida como o uso de medicamentos anti-hipertensivos ou duas ou mais leituras de pressão arterial excedendo 140/90 mmHg. Diabetes mellitus (DM) foi definida como um nível de glicose sérica em jejum excedendo 126 mg/dL ou tratamento atual para diabetes. Tabagismo foi definido como uso ativo de tabaco no momento da inscrição no estudo. Doença renal crônica (DRC) foi definida como creatinina de >1,5 mg/dL ou taxa de filtração glomerular (TFG) de <60 mL/min.

O protocolo do estudo foi aprovado pelo comitê de ética local seguindo as diretrizes da Declaração de Helsinque. A necessidade de consentimento informado por escrito foi dispensada devido à natureza retrospectiva e observacional do estudo.

### Avaliação de angiografia coronária

Imagens de angiografia coronária foram obtidas em múltiplas projeções para as artérias coronárias esquerda e direita, enxertos arteriais e EVSs usando um sistema digital para análise quantitativa. A aortografia foi realizada quando a visualização dos EVSs foi inadequada. A interpretação dos angiogramas coronários foi realizada de forma independente por dois cardiologistas intervencionistas experientes que estavam cegos para as características do paciente. A presença de estenose de 50% no EVS foi considerada indicativa de doença do EVS. Os pacientes foram categorizados em dois grupos com base na presença ou ausência de doença do EVS.

### Sistema de Escore de Nápoles

O NPS compreende quatro componentes: 1) a razão neutrófilo/linfócito (RNL), 2) a razão linfócito/monócito (RLM), 3) nível de colesterol total e 4) nível de albumina sérica. Uma pontuação de 1 é atribuída respectivamente se o nível de albumina sérica estiver abaixo de 40 g/L, o nível de colesterol total for 180 mg/dL ou menor, o RNL exceder 2,96 ou o RLM for 4,44 ou menos. Caso contrário, cada componente recebe uma pontuação de 0,6. Assim, as pontuações totais possíveis variam de 0 a 4 (Figura Suplementar).

### Análise estatística

A análise estatística foi realizada com o software R (R Foundation, Viena, Áustria) e JAMOVI v.2.3.21 (JAMOVI Project, Sydney, Austrália). A normalidade da distribuição dos dados foi analisada com o teste de Kolmogorov–Smirnov. As variáveis contínuas são apresentadas como medianas e intervalos interquartis. As variáveis categóricas são apresentadas como números e porcentagens e o teste qui-quadrado de Pearson ou exato de Fisher foi usado para avaliar associações. Como todas as variáveis contínuas exibiram distribuição anormal, as comparações de grupo foram realizadas usando o teste U de Mann–Whitney. Análise de regressão logística univariada e multivariada foi realizadapara identificar preditores de degeneração do EVS. Modelos estatísticos foram estabelecidos com base no raciocínio clínico. Valores de p<0,05 foram aceitos como estatisticamente significativos.

## Resultados

A população do estudo consistiu em 702 pacientes, incluindo 269 (38,3%) pacientes com EVSs degenerativas e 433 (61,7%) pacientes sem EVSs degenerativas (Tabela 1). HT, DM e DRC foram mais prevalentes entre pacientes com EVSs degenerativas em comparação com aqueles sem degeneração. O grupo com degeneração do EVS teve maiores taxas de IAMCSST e não-IAMCSST, mas uma menor taxa de angina de peito estável. A doença cerebrovascular foi mais frequente no grupo com degeneração do EVS, e a fração de ejeção mediana foi menor. A fibrilação atrial foi mais comum no grupo com degeneração do EVS, e o intervalo de tempo mediano desde a CRM foi maior. O número de EVSs foi maior no grupo com degeneração do EVS. Além disso, as taxas de uso de inibidores da ECA e estatinas foram menores, enquanto o uso de insulina foi maior no grupo com degeneração do EVS. Essas descobertas destacam associações demográficas e clínicas significativas com a degeneração do EVS em pacientes após cirurgia de CRM.

**Tabela 1:** – Características demográficas e clínicas dos pacientes de acordo com a falha do EVS

Variáveis	Degeneração EVS (+) n=269 (38,3%)	Degeneração EVS (-) n=433 (61,7%)	p
Idade (anos)	66 (59-71)	65 (60-73)	0,782
Gênero (masculino), n (%)	222 (82,5)	369 (85,2)	0,342
HT, n (%)	238 (89,5)	326 (75,3)	<0,001*
DM, n (%)	153 (56,9)	148 (34,2)	<0,001*
DRC, n (%)	64 (23,8)	56 (12,9)	<0,001*
APE	84 (31,5)	251 (58)	<0,001*
IAMCSST	21 (7,8)	11 (2,6)	0,001*
Não-IAMCSST	164(61)	171(39,4)	<0,001*
Doença cerebrovascular, n (%)	31 (11,5)	24 (5,5)	0,004*
FE (%)	55 (40-65)	65 (55-65)	<0,001*
Fibrilação atrial, n (%)	39 (14,5)	33 (7,6)	0,004*
Intervalo de tempo desde CRM (ano)	7 (5-9,25)	4 (2-6)	<0,001*
Número de EVSs	3 (2-3)	2 (2-3)	<0,001*
Uso de ácido acetilsalicílico, n (%)	250 (92,9)	413 (95,4)	0,169
Uso de betabloqueadores, n (%)	257 (95,5)	402 (93,5)	0,256
Uso de IECA, n (%)	178 (66,7)	341 (78,8)	<0,001*
Uso de antidiabéticos orais, n (%)	71 (26,8)	98 (22,6)	0,213
Uso de insulina, n (%)	74 (27,8)	73 (16,9)	<0,001*
Uso de estatina, n (%)	185 (68,8)	360 (83,1)	<0,001*

EVS: enxerto de veia safena; HT: hipertensão; DM: diabetes mellitus; DRC: doença renal crônica; APE: angina de peito estável; IAMCSST: infarto do miocárdio com supradesnivelamento do segmento ST; EF: fração de ejeção; CRM: cirurgia de revascularização do miocárdio; IECA: inibidor da enzima conversora da angiotensina.

**Figura 2:**
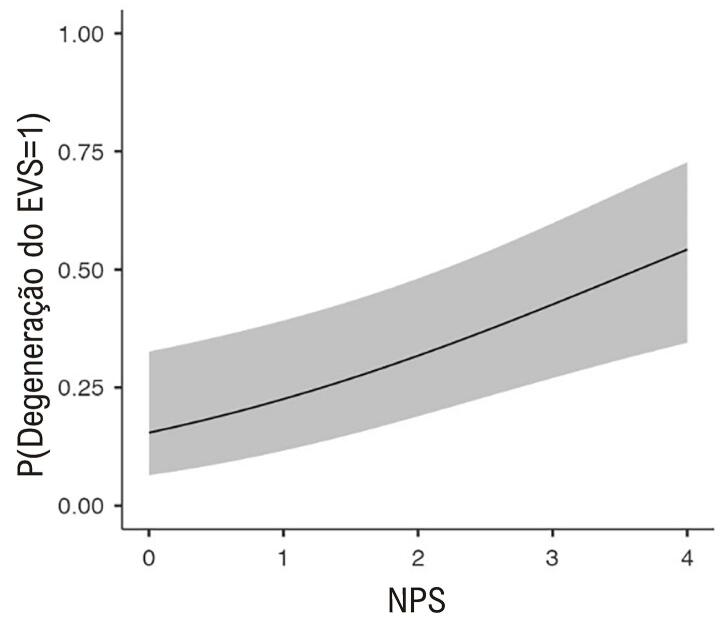
– Box plot da variável escore prognóstico de Nápoles (NPS) em pacientes com e sem degeneração de enxerto de veia safena (EVS).

Na comparação dos achados laboratoriais de acordo com a degeneração do EVS, foram observadas diferenças significativas entre os grupos (Tabela 2). Pacientes com degeneração do EVS apresentaram contagens mais baixas de glóbulos brancos, neutrófilos e linfócitos, juntamente com níveis mais baixos de hemoglobina. Eles também tinham GFR estimada menor, níveis de creatinina mais altos, níveis de albumina mais baixos e NPS mais alto. O gráfico de caixa do NPS em pacientes com e sem degeneração do EVS é mostrado na Figura 2.

**Tabela 2:** – Comparação dos achados laboratoriais dos grupos com base na degeneração de EVSs

Variáveis	Degeneração EVS (+) n=269 (38,3%)	Degeneração EVS (-) n=433 (61,7%)	p
Leucócitos (103/µL)	8,4 (5,9-10,5)	9,1 (6,9-11,6)	0,004*
Neutrófilos	5.1 (3.8-7.1)	6,1 (3,8-7,4)	0,024*
Linfócitos	1,3 (0,9-2,5)	2.1 (1,7-2,5)	<0,001*
Monócitos	0,7 (0,5-1)	0,7 (0,5-0,9)	0,278
Hemoglobina (g/dL)	13.4 (12.1-14)	13,7 (12,6-14,8)	<0,001*
Contagem de plaquetas (103/µL)	243 (194-271)	238 (190-281)	0,279
Colesterol total (mg/dL)	187 (155-205)	189 (157,6-214,2)	0,872
Triglicerídeos (mg/dL)	137 (97-199)	145 (114-238)	0,012
HDL-C (mg/dL)	41 (37-48)	39 (37-49)	0,534
LDL-C (mg/dL)	107 (88-135)	104 (82-138)	0,309
TFGe (mL/min/1,73 m2)	64,9 (37,7-97)	77 (65,8-89)	<0,001*
Creatinina (mg/dL)	1,05 (0,86-1,97)	1 (0,89-1,160)	0,002*
Ácido úrico	6 (5,5-6,7)	5,7 (5,4-6,5)	0,913
Albumina (g/dL)	3.2 (3.-3.7)	3,9 (3,6-4,4)	<0,001*
PCR	15 (4-42,5)	10 (3-40)	0,004
NPS	3 (2-4)	2 (1-3)	<0,001*

EVS: enxerto de veia safena; HDL-C: colesterol de lipoproteína de alta densidade; LDL-C: colesterol de lipoproteína de baixa densidade; TFGe: taxa de filtração glomerular estimada; PCR: proteína C-reativa; NPS: escore prognóstico de Nápoles.

**Figura 3:**
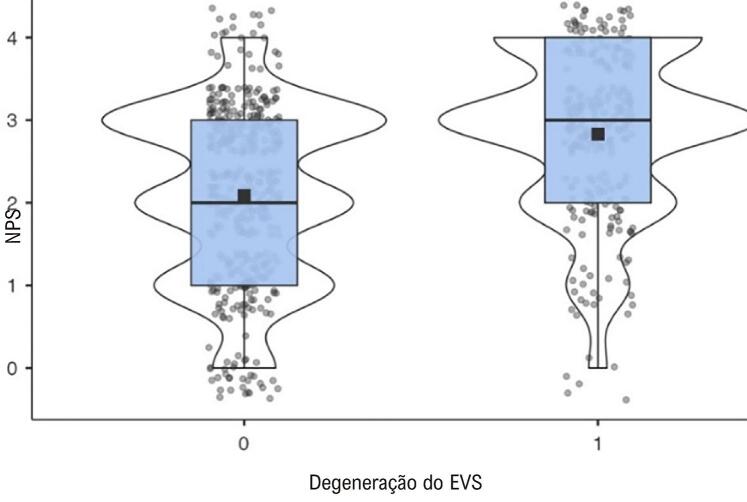
– Gráfico da média marginal mostrando a relação entre o escore prognóstico de Nápoles (NPS) e a probabilidade de degeneração do enxerto de veia safena (EVS).

**Figura 4:**
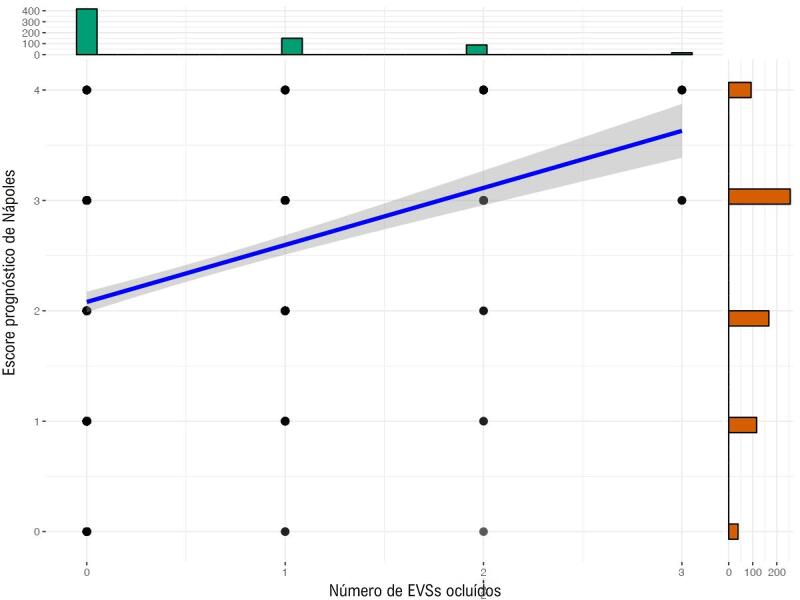
– Gráfico de dispersão mostrando a relação entre o escore prognóstico de Nápoles (NPS) e o número de enxertos de veia safena (EVS) ocluídos.

Na análise multivariada dos preditores de falha do EVS, diversas variáveis foram encontradaspara ter associações significativas (Tabela 3). TH e DRC foram preditores significativos, enquanto o uso de estatina foi associado a um risco reduzido de falha de EVS. O NPS também foi um preditor significativo. Além disso, tanto o intervalo de tempo desde CRM quanto o número de EVSs foram significativamente associados à falha de EVS. A Figura 3 mostra um gráfico de média marginal representando a relação entre o NPS e a probabilidade de degeneração do EVS. Além disso, a Figura 4 apresenta um gráfico de dispersão ilustrando a relação entre o NPS e o número de EVSs ocluídos.

**Tabela 3:** – Análise univariada e multivariada para predição da doença do EVS

Variáveis	Análise multivariada			
	p	OR	Intervalo de confiança de 95%	
			Mais baixo	Superior
Idade (anos)	0,227	0,983	0,956	1.011
Gênero (masculino)	0,113	1.742	0,878	3.456
Hipertensão	0,021	2.344	1.137	4.833
Diabetes mellitus	0,109	1.630	0,897	2.964
Doença cerebrovascular	0,394	0,670	0,267	1.683
Doença renal crônica	0,002*	3.337	1.554	7.168
Fibrilação atrial	0,708	0,867	0,410	1.832
Fração de ejeção	0,795	0,997	0,979	1.017
Uso de IECA	0,110	0,604	0,326	1.121
Uso de insulina	0,356	0,700	0,328	1.494
Uso de estatina	0,006*	0,434	0,239	0,789
Hemoglobina	0,058	0,827	0,679	1.006
Triglicerídeos	0,126	0,997	0,994	1.004
TFGe	0,697	0,998	0,989	1.008
PCR	0,171	1.005	0,998	1.011
NPS	0,001*	1.596	1.198	2.125
Intervalo de tempo desde a CRM (anos)	<0,001*	1.138	1.213	1.432
Número de EVSs	<0,001*	2.708	1.902	3.855

EVS: enxerto de veia safena; OR: razão de chances; IECA: inibidores da enzima de conversão da angiotensina; TFGe: taxa de filtração glomerular estimada; PCR: proteína C-reativa; NPS: escore prognóstico de Nápoles; CRM: cirurgia de revascularização do miocárdio.

## Discussão

Neste estudo, investigamos a significância prognóstica do NPS em pacientes com EVS. Este estudo é o primeiro na literatura a avaliar os efeitos prognósticos do NPS em casos de doença do EVS. Nossas descobertas revelaram que o NPS pode servir como um preditor independente da doença do EVS juntamente com o intervalo de tempo desde CRM e o número de EVSs. Além disso, HT e DRC foram positivamente associados à falha do EVS, enquanto o uso de estatina foi identificado como um preditor independente negativo.

Para pacientes selecionados, a cirurgia de CRM serve como uma abordagem terapêutica eficaz para aliviar os sintomas da doença cardíaca isquêmica, melhorando a qualidade de vida, aumentando a tolerância ao exercício e melhorando as taxas de sobrevivência. Condutos arteriais, mais notavelmente a artéria mamária interna esquerda, bem como condutos venosos safenos, têm sido tradicionalmente utilizados em procedimentos de CRM. Embora os EVSs sejam comumente empregados em CRM, houve uma inclinação recente em direção ao emprego de enxertos arteriais devido às suas taxas de permeabilidade superiores. A taxa de permeabilidade de 10 anos para EVSs é de 61%, enquanto para a artéria mamária interna é de 85%.^11^ Devido a disparidades estruturais e funcionais, os EVSs são consideravelmente mais vulneráveis a eventos trombóticos e ao desenvolvimento de hiperplasia intimal, um precursor da aterosclerose, desencadeado por lesão endotelial e metabolismo lipídico.^12,13^ Estudos identificaram vários fatores predisponentes para doença do EVS, incluindo técnica cirúrgica, diâmetro do vaso nativo, gravidade da estenose proximal, idade do enxerto, HT, DM, tabagismo e hiperlipidemia.^3^ Em nosso estudo, a idade do enxerto e a HT também foram identificadas como fatores predisponentes para doença do EVS, consistentes com achados anteriores.

Vários estudos avaliaram o efeito das estatinas na permeabilidade do EVS. Por exemplo, em 1997, o *Post Coronary Artery Bypass Graft Trial* demonstrou que uma dose maior de lovastatina foi associada a menos progressão da aterosclerose do EVS.^14^ Da mesma forma, um estudo recente de Gaudina et al. descobriu que as estatinas foram associadas a um efeito protetor contra a falha do EVS.^15^ A *American Heart Association* também recomenda iniciar a terapia com estatina no período pré-operatório e retomar seu uso logo após a operação.^16^ Nosso estudo confirma ainda mais o forte efeito protetor das estatinas contra a falha do EVS. As estatinas reduzem o estresse oxidativo vascular em EVSs, melhoram a biodisponibilidade do óxido nítrico e diminuem a inflamação vascular, todos fatores críticos na prevenção da falha do EVS.^17^ Além disso, as estatinas exercem efeitos antitrombóticos e anti-inflamatórios sistêmicos, contribuindo para seus benefícios protetores gerais para pacientes submetidos a CRM.^18^

A falha do EVSs após CRM é uma preocupação significativa devido à sua associação com eventos adversos. Pesquisas anteriores exploraram extensivamente a permeabilidade de EVS, identificando plaquetas e suas funções como principais contribuintes para esse processo. Steele et al. demonstraram uma correlação entre a sobrevivência plaquetária encurtada e a oclusão do enxerto, enquanto outro estudo descobriu que os níveis de plaquetcrit eram preducionista da doença do EVS.^19,20^ Além disso, níveis elevados de largura de distribuição plaquetária foram observados em pacientes com doença do EVS.^21^ Yayla et al. relataram uma razão plaquetas-linfócitos (RPL) significativamente maior em pacientes com doença do EVS em comparação com aqueles com EVSs patentes, com a RPL sendo independentemente associada à doença do EVS mesmo após o ajuste para outros fatores de risco.^22^ Da mesma forma, Oksuz et al. indicaram que a RLM poderia oferecer insights valiosos para avaliação de risco relacionada à doença do EVS em pacientes submetidos a CRM.^23^ Embora uma relação entre os níveis de ácido úrico e a permeabilidade do EVS tenha sido previamente identificada, Oksuz et al. identificaram a razão ácido úrico-albumina como um preditor independente da doença do EVS, sugerindo sua utilidade potencial na previsão da doença do EVS em pacientes com CRM submetidos a intervenção coronária percutânea eletiva.^24,25^ Doğan et al. também demonstrou que a RNL estava independentemente associada à doença do EVS.^26^ Essas descobertas destacam a importância de vários parâmetros hematológicos e seus papéis potenciais na estratificação de risco e no gerenciamento de pacientes com doença do EVS após CRM.

Estudos anteriores claramente vincularam a desnutrição a resultados cardiovasculares adversos, com a desnutrição aumentando o risco de degeneração do enxerto ao prejudicar a integridade vascular e exacerbando a inflamação.^27,28^ A desnutrição enfraquece a função vascular, reduz a saúde endotelial e atrasa a cura, tornando os EVSs mais propensos à falha. Além disso, a resposta inflamatória, intensificada pela desnutrição, acelera o estresse oxidativo e a deterioração do enxerto.^29,30^ Diante disso, a relação entre a degeneração do EVS e a desnutrição destaca a importância da avaliação nutricional e da intervenção em pacientes submetidos à CRM. Abordar a desnutrição por meio de terapias direcionadas pode melhorar a permeabilidade do enxerto e reduzir complicações, levando, em última análise, a melhores resultados clínicos.

O NPS serve como um instrumento valioso para avaliar os níveis de inflamação e nutrição, compreendendo os parâmetros de RNL, RLM, colesterol total e nível de albumina sérica. Inicialmente explorado no contexto de malignidades gastrointestinais,^31,32^ o NPS ganhou mais atenção nos últimos anos dentro do contexto de IAMCSST e HF. Birdal et al. revelaram uma correlação inversa entre o NPS e a fração de ejeção do ventrículo esquerdo na alta emPacientes com IAMCSST.^33^ Saylik et al. associaram independentemente o NPS com mortalidade a longo prazo entre pacientes com IAMCSST submetidos à intervenção coronária percutânea primária.^10^ Da mesma forma, Erdogan et al. observaram associações entre o NPS e resultados hospitalares, bem como eventos pós-alta em pacientes com IAMCSST.^34^ Outro estudo recente de Saygı et al. ilustrou que o NPS poderia prever independentemente a mortalidade hospitalar em casos de IAMCSST.^35^ Além disso, o NPS demonstrou ser um preditor independente de pontuações SYNTAX intermediárias a altas em pacientes com IAMCSST.^36^ Investigações recentes se expandiram para incluir explorações do valor prognóstico do NPS em pacientes com IC. Kilic et al. identificaram uma correlação robusta entre o NPS e a mortalidade na IC, enquanto Erdogan et al. demonstraram ligações entre o NPS e as taxas de mortalidade, juntamente com os riscos de re-hospitalização em pacientes com IC descompensada.^8,9^ Além disso, um estudo recente de Arugaslan et al. revelou uma associação entre NPS e resultados adversos em pacientes com HT arterial pulmonar.^37^

Estudos também foram conduzidos sobre a relação entre o NPS e outras doenças cardiovasculares. Por exemplo, se descobriu que o NPS estava correlacionado com mortalidade por todas as causas e amputação após terapia endovascular em pacientes com doença arterial periférica.^38^ Pay et al. mostraram que o NPS pode ter o potencial de prever mortalidade em longo prazo entre pacientes com embolia pulmonar aguda.^39^ Além disso, há estudos demonstrando a relação entre prognóstico e NPS em pacientes submetidos à substituição da válvula aórtica transcateter (TAVI). Çetin et al. descobriram que o NPS serve como um preditor confiável de mortalidade em um ano em pacientes com estenose aórtica grave submetidos a TAVI.^40^ Da mesma forma, Demirci et al. revelaram que o NPS forneceu informações prognósticas valiosas para mortalidade por todas as causas em longo prazo em pacientes com estenose aórtica grave submetidos a TAVI.^41^

No entanto, embora estudos tenham mostrado a relação entre RNL e RLM como componentes da permeabilidade do NPS e do EVS,^18,20^ há uma falta de evidências diretas ligando o NPS com a doença do EVS na literatura. Em nosso estudo, observamos um aumento significativo na doença do EVS entre pacientes com altos valores de NPS. Esse achado pode ser atribuído às semelhanças entre os parâmetros do NPS e as causas da oclusão do EVS, como inflamação e desnutrição. Portanto, o NPS pode ser útil na avaliação e previsão da permeabilidade do EVS em pacientes submetidos à CRM.

Este estudo tem várias limitações que precisam ser reconhecidas. Primeiro, seu desenho retrospectivo limita a avaliação do NPS, pois apenas os valores de NPS de admissão foram avaliados sem acompanhamento.avaliações. Segundo, a generalização de nossas descobertas é restrita devido à natureza de centro único do estudo, garantindo a validação por meio de estudos multicêntricos. Terceiro, o tamanho de nossa amostra foi relativamente pequeno, enfatizando a necessidade de validação adicional por meio de estudos prospectivos com coortes maiores para confirmar e generalizar nossos resultados. A inclusão de pacientes com síndrome coronariana aguda pode afetar a validade do NPS, pois as respostas inflamatórias e metabólicas nesses pacientes diferem daquelas com doença arterial coronariana estável, influenciando potencialmente a precisão da pontuação.

## Conclusão

O NPS, uma ferramenta valiosa para avaliar inflamação e estado nutricional, pode oferecer insights sobre a permeabilidade do EVS após cirurgia de CRM. Pacientes com NPS elevado após CRM devem passar por monitoramento cuidadoso para o início da doença do EVS. Além disso, a avaliação pré-operatória do NPS pode auxiliar na determinação da duração ideal da terapia antiplaquetária dupla e na defesa do uso de enxertos arteriais com taxas de permeabilidade superiores.

## References

[1] Vries MR, Simons KH, Jukema JW, Braun J, Quax PH (2016). Vein Graft Failure: From Pathophysiology to Clinical Outcomes. Nat Rev Cardiol.

[2] Owens CD (2010). Adaptive Changes in Autogenous Vein Grafts for Arterial Reconstruction: Clinical Implications. J Vasc Surg.

[3] Motwani JG, Topol EJ (1998). Aortocoronary Saphenous Vein Graft Disease: Pathogenesis, Predisposition, and Prevention. Circulation.

[4] Parang P, Arora R (2009). Coronary Vein Graft Disease: Pathogenesis and Prevention. Can J Cardiol.

[5] Bourassa MG, Fisher LD, Campeau L, Gillespie MJ, McConney M, Lespérance J (1985). Long-Term Fate of Bypass Grafts: The Coronary Artery Surgery Study (CASS) and Montreal Heart Institute experiences. Circulation.

[6] Galizia G, Lieto E, Auricchio A, Cardella F, Mabilia A, Podzemny V (2017). Naples Prognostic Score, Based on Nutritional and Inflammatory Status, is an Independent Predictor of Long-Term Outcome in Patients Undergoing Surgery for Colorectal Cancer. Dis Colon Rectum.

[7] Galizia G, Auricchio A, Vita F, Cardella F, Mabilia A, Basile N (2019). Inflammatory and Nutritional Status is a Predictor of Long-Term Outcome in Patients Undergoing Surgery for Gastric Cancer. Validation of the Naples Prognostic Score. Ann Ital Chir.

[8] Kiliç O, Suygun H, Mustu M, Karadeniz FO, Ozer SF, Senol H (2023). Is the Naples Prognostic Score Useful for Predicting Heart Failure Mortality. Kardiologiia.

[9] Erdogan A, Genc O, Inan D, Yildiz U, Balaban I, Guler Y (2023). Impact of Naples Prognostic Score on Midterm All-Cause Mortality in Patients with Decompensated Heart Failure. Biomark Med.

[10] Saylik F, Çinar T, Selçuk M, Akbulut T, Hayiroglu MI, Tanboga IH (2024). Evaluation of Naples Score for Long-Term Mortality in Patients with ST-Segment Elevation Myocardial Infarction Undergoing Primary Percutaneous Coronary Intervention. Angiology.

[11] Goldman S, Zadina K, Moritz T, Ovitt T, Sethi G, Copeland JG (2004). Long-Term Patency of Saphenous Vein and Left Internal Mammary Artery Grafts after Coronary Artery Bypass Surgery: Results from a Department of Veterans Affairs Cooperative Study. J Am Coll Cardiol.

[12] Nwasokwa ON (1995). Coronary Artery bypass Graft Disease. Ann Intern Med.

[13] Fitzgibbon GM, Kafka HP, Leach AJ, Keon WJ, Hooper GD, Burton JR (1996). Coronary Bypass Graft Fate and Patient Outcome: Angiographic Follow-Up of 5,065 Grafts Related to Survival and Reoperation in 1,388 Patients during 25 Years. J Am Coll Cardiol.

[14] Post Coronary Artery Bypass Graft Trial Investigators (1997). The Effect of Aggressive Lowering of Low-Density Lipoprotein Cholesterol Levels and Low-Dose Anticoagulation on Obstructive Changes in Saphenous-Vein Coronary-Artery Bypass Grafts. N Engl J Med.

[15] Gaudino M, Sandner S, An KR, Dimagli A, Franco A, Audisio K (2023). Graft Failure after Coronary Artery Bypass Grafting and Its Association with Patient Characteristics and Clinical Events: A Pooled Individual Patient Data Analysis of Clinical Trials with Imaging Follow-Up. Circulation.

[16] Kulik A, Ruel M, Jneid H, Ferguson TB, Hiratzka LF, Ikonomidis JS (2015). Secondary Prevention after Coronary Artery Bypass Graft Surgery: A Scientific Statement from the American Heart Association. Circulation.

[17] Zhou Q, Liao JK (2009). Statins and Cardiovascular Diseases: From Cholesterol Lowering to Pleiotropy. Curr Pharm Des.

[18] Veillard NR, Braunersreuther V, Arnaud C, Burger F, Pelli G, Steffens S (2006). Simvastatin Modulates Chemokine and Chemokine Receptor Expression by Geranylgeranyl Isoprenoid Pathway in Human Endothelial Cells and Macrophages. Atherosclerosis.

[19] Steele P, Battock D, Pappas G, Genton E (1976). Correlation of Platelet Survival Time with Occlusion of Saphenous Vein Aorto-Coronary Bypass Grafts. Circulation.

[20] Akpinar I, Sayin MR, Gursoy YC, Karabag T, Kucuk E, Buyukuysal MC (2014). Plateletcrit. A Platelet Marker Associated with Saphenous Vein Graft Disease. Herz.

[21] Ege MR, Guray U, Guray Y, Acikgoz S, Demirkan B (2013). Platelet Distribution Width and Saphenous Vein Disease in Patients after CABG. Association with Graft Occlusion. Herz.

[22] Yayla Ç, Canpolat U, Akyel A, Yayla KG, Yilmaz S, Açikgöz SK (2016). Association between Platelet to Lymphocyte Ratio and Saphenous Vein Graft Disease. Angiology.

[23] Oksuz F, Elcik D, Yarlioglues M, Duran M, Ozturk S, Celik IE (2017). The Relationship between Lymphocyte-to-Monocyte Ratio and Saphenous Vein Graft Patency in Patients with Coronary Artery Bypass Graft. Biomark Med.

[24] Tavil Y, Sen N, Hizal F, Açikgöz SK, Tasoglu I, Topal S (2008). Relationship between Elevated Levels of Serum Uric Acid and Saphenous Vein Graft Disease. Turk Kardiyol Dern Ars.

[25] Oksuz F, Yarlioglues M, Karacali K, Erat M, Celik IE, Duran M (2024). Relationship between Uric Acid to Albumin Ratio and Saphenous Vein Graft Disease in Patients with Coronary Artery Bypass Graft. Coron Artery Dis.

[26] Dogan M, Akyel A, Cimen T, Öksüz F, Celik IE, Aytürk M (2015). Relationship between Neutrophil-to-Lymphocyte Ratio and Saphenous Vein Graft Disease in Patients with Coronary Bypass. Clin Appl Thromb Hemost.

[27] Keskin M, Ipek G, Aldag M, Altay S, Hayiroglu MI, Börklü EB (2018). Effect of Nutritional Status on Mortality in Patients Undergoing Coronary Artery Bypass Grafting. Nutrition.

[28] Sciatti E, Lombardi C, Ravera A, Vizzardi E, Bonadei I, Carubelli V (2016). Nutritional Deficiency in Patients with Heart Failure. Nutrients.

[29] Gomes F, Emery PW, Weekes CE (2016). Risk of Malnutrition is an Independent Predictor of Mortality, Length of Hospital Stay, and Hospitalization Costs in Stroke Patients. J Stroke Cerebrovasc Dis.

[30] Dong M, Cheng J, Gong L, Xiao Y, Shao S, Song J (2023). Malnutrition Predicts Adverse Outcomes after Transcatheter Aortic Valve Replacement: A Systematic Review and Meta-Analysis. Anatol J Cardiol.

[31] Nakagawa N, Yamada S, Sonohara F, Takami H, Hayashi M (2020). Clinical Implications of Naples Prognostic Score in Patients with Resected Pancreatic Cancer. Ann Surg Oncol.

[32] Miyamoto Y, Hiyoshi Y, Daitoku N, Okadome K, Sakamoto Y, Yamashita K (2019). Naples Prognostic Score Is a Useful Prognostic Marker in Patients with Metastatic Colorectal Cancer. Dis Colon Rectum.

[33] Birdal O, Pay L, Aksakal E, Yumurtas AÇ, Çinier G, Yücel E (2024). Naples Prognostic Score and Prediction of Left Ventricular Ejection Fraction in STEMI Patients. Angiology.

[34] Erdogan A, Genc O, Ozkan E, Goksu MM, Ibisoglu E, Bilen MN (2023). Impact of Naples Prognostic Score at Admission on In-Hospital and Follow-Up Outcomes among Patients with ST-Segment Elevation Myocardial Infarction. Angiology.

[35] Saygi M, Tanalp AC, Tezen O, Pay L, Dogan R, Uzman O (2024). The Prognostic Importance of the Naples Prognostic Score for In-Hospital Mortality in Patients with ST-Segment Elevation Myocardial Infarction. Coron Artery Dis.

[36] Oner E, Kahraman S, Agus HZ, Guner A, Dogan AC, Yildiz MM (2023). Naples Score is Associated with SYNTAX Score in Patients with ST-Segment Elevation Myocardial Infarction. Coron Artery Dis.

[37] Arugaslan E, Kalayci S, Tufekcioglu O (2023). Naples Prognostic Score and Clinical Outcomes in Pulmonary Arterial Hypertension Patients. Sisli Etfal Hastan Tip Bul.

[38] Artac I, Karakayali M, Omar T, Ilis D, Arslan A, Sahin MH (2024). Predictive Value of the Naples Prognostic Score on Long-Term Outcomes in Patients with Peripheral Artery Disease Revascularized via Percutaneous Intervention. Ann Vasc Surg.

[39] Pay L, Çetin T, Keskin K, Dereli S, Tezen O, Yumurtas AÇ (2024). Evaluation of Naples Prognostic Score to Predict Long-Term Mortality in Patients with Pulmonary Embolism. Biomark Med.

[40] Çetin ZG, Balun A, Çiçekçioglu H, Demirtas B, Yigitbasi MM, Özbek K (2023). A Novel Score to Predict One-Year Mortality after Transcatheter Aortic Valve Replacement, Naples Prognostic Score. Medicina.

[41] Demirci G, Aslan S, Güner A, Demir AR, Erata YE, Türkmen I (2024). Clinical Implication of the Naples Prognostic Score on Transcatheter Aortic Valve Replacement in Patients with Severe Aortic Stenosis. Catheter Cardiovasc Interv.

