# Supplementary material for: Relação entre o Escore Prognóstico de Nápoles e a Doença do Enxerto de Veia Safena após Cirurgia de Revascularização do Miocárdio
Source: Arq Bras Cardiol. 2025 May 15;122(5):e20240519. [Article in Portuguese] doi: 10.36660/abc.20240519 (PMC12129478; doi:10.36660/abc.20240519)
Supplement: Supplementary file 1 [file 0066-782x-abc-122-5-e20240519-suppl1.pdf]

Supplementary Figure. Calculation of Naples prognostic score

Cut-off values of variables	Point
**Serum albumin (mg/dl)**	
<4	1
≥4	0
**Total-C (mg/dl)**	
≤180	1
>180	0
**NLR**	
>2.96	1
≤2.96	0
**LMR**	
≤4.44	1
>4.44	0
