# Supplementary material for: Relação entre o Escore Prognóstico de Nápoles e a Doença do Enxerto de Veia Safena após Cirurgia de Revascularização do Miocárdio
Source: Arq Bras Cardiol. 2025 May 15;122(5):e20240519. [Article in Portuguese] doi: 10.36660/abc.20240519 (PMC12129478; doi:10.36660/abc.20240519)
Supplement: Translation

# Translation: Relationship Between the Naples Prognostic Score and Saphenous Vein Graft Disease after Coronary Artery Bypass Grafting Surgery

**DOI:** 10.36660/abc.20240519i

**Authors:** Ahmet Karaduman, Cemalettin Yılmaz, Muhammet Mucahit Tiryaki, İsmail Balaban, Mustafa Ferhat Keten, Tuba Unkun, Servet İzci, Suleyman Çağan Efe, Elnur Alizade

**Affiliations:** 1 Türkiye Department of Cardiology, Kartal Kosuyolu Research and Education Hospital, Istanbul – Türkiye; 2 Türkiye Department of Cardiology, Mus State Hospital, Mus – Türkiye

**Keywords:** Myocardial Revascularization, Saphenous Vein, Inflammation, Atherosclerosis

## Abstract

**Background:**

Saphenous vein graft (SVG) patency remains a challenge in cases of coronary artery disease following coronary artery bypass grafting (CABG) surgery. The Naples prognostic score (NPS) constitutes a novel scoring system designed to assess both nutritional status and inflammation.

**Objectives:**

Our study aimed to explore the association between the NPS and SVG disease in patients with a previous history of CABG surgery.

**Methods:**

A total of 702 patients who had undergone CABG surgery and underwent coronary angiography were reviewed retrospectively. SVG disease was defined as the presence of ≥50% stenosis in at least one SVG. Patients were categorized into two groups based on the presence or absence of SVG disease. Values of p<0.05 were accepted as statistically significant.

**Results:**

The study population consisted of 702 patients, with 269 (38.3%) having degenerative SVGs and 433 (61.7%) without degenerative SVGs. The NPS was higher in the group with saphenous vein degeneration and emerged as a significant predictor of SVG disease (OR: 1.596, 95% CI: 1.198-2.125, p=0.001). Additionally, hypertension (OR: 2.344, 95% CI: 1.137-4.833, p=0.02), chronic kidney disease (OR: 3.337, 95% CI: 1.554-7.168, p=0.002), statin usage (OR: 0.434, 95% CI: 0.239-0.789, p=0.006), time interval since CABG (OR: 1.138, 95% CI: 1.213-1.432, p<0.001), and number of SVGs (OR: 2.708, 95% CI: 1.902-3.855, p<0.001) were significant predictors of SVG disease.

**Conclusion:**

The NPS, a useful tool for assessing inflammation and nutritional status, could provide valuable information about the patency of SVGs following CABG surgery. Patients with elevated NPS after CABG should undergo careful monitoring for the development of SVG disease.

## Introduction

Coronary artery bypass grafting (CABG) surgery is an effective therapeutic approach utilized for many years to alleviate angina episodes, enhance quality of life, and extend the lifespan of patients with coronary artery disease. Both arterial and venous grafts are viable options for this procedure. However, compared to arterial grafts, saphenous vein grafts (SVGs) have lower patency rates, particularly in contrast to the internal mammary artery. Approximately 12% of SVGs experience occlusion within one month following surgery, with the occlusion rate escalating to 40% by the tenth year after CABG surgery.^1,2^ Various mechanisms have been postulated for SVG disease, including thrombosis, intimal hyperplasia, and atherosclerosis.^3,4^ In addition to established atherosclerosis risk factors, factors such as SVG age and the native vein diameter in CABG procedures may also contribute to the development of SVG pathologies.^5^ The significance of each mechanism may vary among patients, prompting research endeavors aimed at identifying risk indicators for SVG disease.

The Naples prognostic score (NPS) constitutes a newly developed scoring system incorporating serum albumin and total cholesterol levels together with the neutrophil-to-lymphocyte ratio (NLR) and lymphocyte-to-monocyte ratio (LMR), aimed at evaluating the nutritional and inflammatory status of patients.^6,7^ Initially validated as a prognostic indicator for patients undergoing colorectal cancer surgery, the NPS has more recently been linked to adverse outcomes in individuals with acute coronary syndrome and heart failure (HF).^8-10^ Despite the successful establishment of associations between patient prognosis and NPS in various cardiovascular disease cohorts, there is a notable lack of research addressing its relevance to SVG patency. Therefore, our study aimed to explore the relationship between NPS and SVG disease in patients with a history of CABG surgery.

## Methods

### Study Population

This retrospective observational study included patients who had previously undergone CABG and subsequently underwent coronary angiography between January 2016 and May 2024. All patients presented with stable angina symptoms and/or positive stress test results or acute coronary syndrome. Clinical, demographic, and laboratory data were collected through a review of medical records. Information regarding the medications used by patients prior to the coronary angiography procedure was also documented. Baseline coronary angiography results were analyzed, and the mean time interval between CABG and baseline coronary angiography was calculated.

The exclusion criteria included significant valvular heart disease, decompensated HF, acute or chronic pulmonary disease, autoimmune disease, infectious or inflammatory disease, left internal mammary artery disease, the use of steroids or anti-inflammatory medications, and a history of hematological disorders or malignancy. After screening for the inclusion and exclusion criteria, 702 patients with SVG were included in the final analysis. The flow chart of the study population is shown in Figure 1. These patients were categorized into two groups based on SVG patency.

**Figure 1:** – CONSORT of the study population.

Hypertension (HT) was defined as the use of antihypertensive drugs or two or more blood pressure readings exceeding 140/90 mmHg. Diabetes mellitus (DM) was defined as a fasting serum glucose level exceeding 126 mg/dL or current treatment for diabetes. Smoking was defined as active tobacco use at the time of study enrollment. Chronic kidney disease (CKD) was defined as creatinine of >1.5 mg/dL or glomerular filtration rate (GFR) of <60 mL/min.

The study protocol was approved by the local ethics committee following the Declaration of Helsinki guidelines. The need for written informed consent was waived due to the retrospective and observational nature of the study.

### Coronary angiography evaluation

Coronary angiography images were obtained in multiple projections for the left and right coronary arteries, arterial grafts, and SVGs using a digital system for quantitative analysis. Aortography was performed when visualization of the SVGs was inadequate. Interpretation of the coronary angiograms was performed independently by two experienced interventional cardiologists who were blinded to patient characteristics. The presence of 50% stenosis in the SVG was considered indicative of SVG disease. Patients were categorized into two groups based on the presence or absence of SVG disease.

### Naples Scoring System

The NPS comprises four components: 1) the neutrophil/lymphocyte ratio (NLR), 2) the lymphocyte/monocyte ratio (LMR), 3) total cholesterol level, and 4) serum albumin level. A score of 1 is respectively assigned if the serum albumin level is below 40 g/L, the total cholesterol level is 180 mg/dL or lower, the NLR exceeds 2.96, or the LMR is 4.44 or less. Otherwise, each component receives a score of 0.6 Thus, total possible scores range from 0 to 4 (Supplementary Figure).

### Statistical analysis

Statistical analysis was performed with R (R Foundation, Vienna, Austria) and JAMOVI v.2.3.21 (JAMOVI Project, Sydney, Australia) software. The normality of the distribution of the data was analyzed with the Kolmogorov–Smirnov test. Continuous variables are presented as medians and interquartile ranges. Categorical variables are presented as numbers and percentages and Pearson’s chi-square or Fisher’s exact test was used to evaluate associations. Since all continuous variables exhibited abnormal distribution, group comparisons were performed using the Mann–Whitney U test. Univariate and multivariate logistic regression analysis was performed to identify predictors of SVG degeneration. Statistical models were established based on clinical rationale. Values of p<0.05 were accepted as statistically significant.

## Results

The study population consisted of 702 patients, including 269 (38.3%) patients with degenerative SVGs and 433 (61.7%) patients without degenerative SVGs (Table 1). HT, DM, and CKD were more prevalent among patients with degenerative SVGs compared to those without degeneration. The SVG degeneration group had higher rates of STEMI and non-STEMI but a lower rate of stable angina pectoris. Cerebrovascular disease was more frequent in the SVG degeneration group, and the median ejection fraction was lower. Atrial fibrillation was more common in the SVG degeneration group, and the median time interval since CABG was longer. The number of SVGs was higher in the SVG degeneration group. Additionally, ACE inhibitor and statin usage rates were lower, while insulin usage was higher in the SVG degeneration group. These findings highlight significant demographic and clinical associations with SVG degeneration in patients following CABG surgery.

**Table 1:** – Demographic and clinical characteristics of patients according to SVG failure

Variables	SVG degeneration (+) n=269 (38.3%)	SVG degeneration (-) n=433 (61.7%)	p
Age (years)	66 (59-71)	65 (60-73)	0.782
Gender (male), n (%)	222 (82.5)	369 (85.2)	0.342
HT, n (%)	238 (89.5)	326 (75.3)	<0.001*
DM, n (%)	153 (56.9)	148 (34.2)	<0.001*
CKD, n (%)	64 (23.8)	56 (12.9)	<0.001*
SAP	84 (31.5)	251 (58)	<0.001*
STEMI	21 (7.8)	11 (2.6)	0.001*
Non-STEMI	164(61)	171(39.4)	<0.001*
Cerebrovascular disease, n (%)	31 (11.5)	24 (5.5)	0.004*
EF (%)	55 (40-65)	65 (55-65)	<0.001*
Atrial fibrillation, n (%)	39 (14.5)	33 (7.6)	0.004*
Time interval since CABG (year)	7 (5-9.25)	4 (2-6)	<0.001*
Number of SVGs	3 (2-3)	2 (2-3)	<0.001*
Acetylsalicylic acid usage, n (%)	250 (92.9)	413 (95.4)	0.169
Betablocker usage, n (%)	257 (95.5)	402 (93.5)	0.256
ACEi usage, n (%)	178 (66.7)	341 (78.8)	<0.001*
Oral anti-diabetic usage, n (%)	71 (26.8)	98 (22.6)	0.213
Insulin usage, n (%)	74 (27.8)	73 (16.9)	<0.001*
Statin usage, n (%)	185 (68.8)	360 (83.1)	<0.001*

SVG: saphenous vein graft; HT: hypertension; DM: diabetes mellitus; CKD: chronic kidney disease; SAP: stable angina pectoris; STEMI: ST-elevation myocardial infarction; EF: ejection fraction; CABG: coronary artery bypass grafting surgery; ACEi: angiotensin-converting enzyme inhibitor.

In the comparison of laboratory findings according to SVG degeneration, significant differences were observed between the groups (Table 2). Patients with SVG degeneration had lower white blood cell, neutrophil, and lymphocyte counts, along with lower hemoglobin levels. They also had lower estimated GFR, higher creatinine levels, lower albumin levels, and higher NPS. The box plot of the NPS in patients with and without SVG degeneration is shown in Figure 2.

**Table 2:** – Comparison of laboratory findings of the groups based on degeneration of SVGs

Variables	SVG degeneration (+) n=269 (38.3%)	SVG degeneration (-) n=433 (61.7%)	p
WBC (10^3^/µL)	8.4 (5.9-10.5)	9.1 (6.9-11.6)	0.004*
Neutrophils	5.1 (3.8-7.1)	6.1 (3.8-7.4)	0.024*
Lymphocytes	1.3 (0.9-2.5)	2.1 (1.7-2.5)	<0.001*
Monocytes	0.7 (0.5-1)	0.7 (0.5-0.9)	0.278
Hemoglobin (g/dL)	13.4 (12.1-14)	13.7 (12.6-14.8)	<0.001*
Platelet count (10^3^/µL)	243 (194-271)	238 (190-281)	0.279
Total cholesterol (mg/dL)	187 (155-205)	189 (157.6-214.2)	0.872
Triglycerides (mg/dL)	137 (97-199)	145 (114-238)	0.012
HDL-C (mg/dL)	41 (37-48)	39 (37-49)	0.534
LDL-C (mg/dL)	107 (88-135)	104 (82-138)	0.309
eGFR (mL/min/1.73 m^2^)	64.9 (37.7-97)	77 (65.8-89)	<0.001*
Creatinine (mg/dL)	1.05 (0.86-1.97)	1 (0.89-1.160)	0.002*
Uric acid	6 (5.5-6.7)	5.7 (5.4-6.5)	0.913
Albumin (g/dL)	3.2 (3.-3.7)	3.9 (3.6-4.4)	<0.001*
CRP	15 (4-42.5)	10 (3-40)	0.004
NPS	3 (2-4)	2 (1-3)	<0.001*

SVG: saphenous vein graft; WBC: white blood cell count; HDL-C: high-density lipoprotein cholesterol; LDL-C: low-density lipoprotein cholesterol; eGFR: estimated glomerular filtration rate; CRP: C-reactive protein; NPS: Naples prognostic score.

**Figure 2:** – A box plot of the Naples prognostic score (NPS) variable in patients with and without saphenous vein graft (SVG) degeneration.

In the multivariate analysis of predictors of SVG failure, several variables were found to have significant associations (Table 3). HT and CKD were significant predictors, while statin usage was associated with a reduced risk of SVG failure. The NPS was also a significant predictor. Additionally, both the time interval since CABG and the number of SVGs were significantly associated with SVG failure. Figure 3 shows a marginal mean plot depicting the relationship between the NPS and the probability of SVG degeneration. Additionally, Figure 4 presents a scatter plot illustrating the relationship between the NPS and the number of occluded SVGs.

**Table 3:** – Univariate and multivariate analysis for prediction of SVG disease

Variables	Multivariate analysis			
	p	OR	95% Confidence interval	
			Lower	Upper
Age (years)	0.227	0.983	0.956	1.011
Gender (male)	0.113	1.742	0.878	3.456
Hypertension	0.021	2.344	1.137	4.833
Diabetes mellitus	0.109	1.630	0.897	2.964
Cerebrovascular disease	0.394	0.670	0.267	1.683
Chronic kidney disease	0.002*	3.337	1.554	7.168
Atrial fibrillation	0.708	0.867	0.410	1.832
Ejection fraction	0.795	0.997	0.979	1.017
ACEi usage	0.110	0.604	0.326	1.121
Insulin usage	0.356	0.700	0.328	1.494
Statin usage	0.006*	0.434	0.239	0.789
Hemoglobin	0.058	0.827	0.679	1.006
Triglycerides	0.126	0.997	0.994	1.004
eGFR	0.697	0.998	0.989	1.008
CRP	0.171	1.005	0.998	1.011
NPS	0.001*	1.596	1.198	2.125
Time interval since CABG (years)	<0.001*	1.138	1.213	1.432
Number of SVGs	<0.001*	2.708	1.902	3.855

SVG: saphenous vein graft; OR: odds ratio; ACEi: angiotensin-converting enzyme inhibitors; eGFR: estimated glomerular filtration rate; CRP: C-reactive protein; NPS: Naples prognostic score; CABG: coronary artery bypass grafting surgery.

**Figure 3:** – The marginal mean graph showing the relationship between Naples prognostic score (NPS) and probability of saphenous vein graft (SVG) degeneration.

**Figure 4:** – A scatter plot showing the relationship between the Naples prognostic score (NPS) and the number of occluded saphenous vein grafts (SVG).

## Discussion

In this study, we investigated the prognostic significance of the NPS in patients with SVG. This study is the first in the literature to evaluate the prognostic effects of the NPS in cases of SVG disease. Our findings revealed that the NPS can serve as an independent predictor of SVG disease alongside the time interval since CABG and the number of SVGs. Additionally, HT and CKD were positively associated with SVG failure, while statin usage was identified as a negative independent predictor.

For selected patients, CABG surgery serves as an efficacious therapeutic approach for alleviating the symptoms of ischemic heart disease, enhancing quality of life, increasing exercise tolerance, and improving survival rates. Arterial conduits, most notably the left internal mammary artery, as well as saphenous venous conduits, have traditionally been utilized in CABG procedures. While SVGs are commonly employed in CABG, there has been a recent inclination towards employing arterial grafts due to their superior patency rates. The 10-year patency rate for SVGs stands at 61%, whereas for the internal mammary artery, it is 85%.^11^ Due to structural and functional disparities, SVGs are considerably more vulnerable to thrombotic events and the development of intimal hyperplasia, a precursor to atherosclerosis, triggered by endothelial injury and lipid metabolism.^12,13^ Studies have identified several predisposing factors for SVG disease, including surgical technique, native vessel diameter, severity of proximal stenosis, graft age, HT, DM, smoking, and hyperlipidemia.^3^ In our study, graft age and HT were also identified as predisposing factors for SVG disease, consistent with previous findings.

Multiple studies have evaluated the effect of statins on SVG patency. For example, in 1997, the Post Coronary Artery Bypass Graft Trial demonstrated that a higher dose of lovastatin was associated with less progression of SVG atherosclerosis.^14^ Similarly, a recent study by Gaudina et al. found that statins were associated with a protective effect against SVG failure.^15^ The American Heart Association also recommends starting statin therapy in the preoperative period and resuming its use early after the operation.^16^ Our study further confirms the strong protective effect of statins against SVG failure. Statins reduce vascular oxidative stress in SVGs, improve nitric oxide bioavailability, and decrease vascular inflammation, all critical factors in preventing SVG failure.^17^ Additionally, statins exert systemic antithrombotic and anti-inflammatory effects, contributing to their overall protective benefits for patients undergoing CABG.^18^

The failure of SVGs following CABG is a significant concern due to its association with adverse events. Previous research has extensively explored SVG patency, identifying platelets and their functions as key contributors to this process. Steele et al. demonstrated a correlation between shortened platelet survival and graft occlusion, while another study found that plateletcrit levels were predictive of SVG disease.^19,20^Additionally, elevated platelet distribution width levels have been observed in patients with SVG disease.^21^ Yayla et al. reported a significantly higher platelet-to-lymphocyte ratio (PLR) in patients with SVG disease compared to those with patent SVGs, with the PLR being independently associated with SVG disease even after adjusting for other risk factors.^22^ Similarly, Oksuz et al. indicated that the LMR could offer valuable insights for risk assessment related to SVG disease in patients undergoing CABG.^23^ Although a relationship between uric acid levels and SVG patency has been previously identified, Oksuz et al. identified the uric acid-to-albumin ratio as an independent predictor of SVG disease, suggesting its potential utility in predicting SVG disease in patients with CABG who undergo elective percutaneous coronary intervention.^24,25^ Dogan et al. also demonstrated that the NLR was independently associated with SVG disease.^26^ These findings highlight the importance of various hematological parameters and their potential roles in the risk stratification and management of patients with SVG disease following CABG.

Previous studies have clearly linked malnutrition to adverse cardiovascular outcomes, with malnutrition increasing the risk of graft degeneration by impairing vascular integrity and exacerbating inflammation.^27,28^ Malnutrition weakens vascular function, reduces endothelial health, and delays healing, making SVGs more prone to failure. Additionally, the inflammatory response, heightened by malnutrition, accelerates oxidative stress and graft deterioration.^29,30^Given this, the relationship between SVG degeneration and malnutrition highlights the importance of nutritional assessment and intervention in patients undergoing CABG. Addressing malnutrition through targeted therapies may improve graft patency and reduce complications, ultimately leading to better clinical outcomes.

The NPS serves as a valuable instrument for evaluating levels of inflammation and nutrition, comprising the parameters of NLR, LMR, total cholesterol, and serum albumin level. Initially explored in the context of gastrointestinal malignancies,^31,32^ the NPS has garnered further attention in recent years within the context of STEMI and HF. Birdal et al. revealed an inverse correlation between the NPS and left ventricular ejection fraction at discharge in STEMI patients.^33^ Saylik et al. independently associated the NPS with long-term mortality among STEMI patients undergoing primary percutaneous coronary intervention.^10^ Similarly, Erdogan et al. observed associations between the NPS and in-hospital outcomes as well as post-discharge events in STEMI patients.^34^ Another recent study by Saygı et al. illustrated that the NPS could independently predict in-hospital mortality in cases of STEMI.^35^ Additionally, the NPS was shown to be an independent predictor of intermediate to high SYNTAX scores in STEMI patients.^36^ Recent investigations have expanded to include explorations of the prognostic value of the NPS in HF patients. Kilic et al. identified a robust correlation between the NPS and mortality in HF, while Erdogan et al. demonstrated links between the NPS and mortality rates along with rehospitalization risks in decompensated HF patients.^8,9^ Furthermore, a recent study by Arugaslan et al. revealed an association between NPS and adverse outcomes in patients with pulmonary arterial hypertension.^37^

Studies have also been conducted on the relationship between the NPS and other cardiovascular diseases. For example, the NPS was found to be correlated with all-cause mortality and amputation following endovascular therapy in patients with peripheral artery disease.^38^ Pay et al. showed that the NPS may have the potential to predict long-term mortality among patients with acute pulmonary embolism.^39^ Additionally, there are studies demonstrating the relationship between prognosis and NPS in patients undergoing transcatheter aortic valve replacement (TAVR). Çetin et al. found that the NPS serves as a reliable predictor of one-year mortality in patients with severe aortic stenosis undergoing TAVR.^40^ Similarly, Demirci et al. revealed that the NPS provided valuable prognostic information for long-term all-cause mortality in patients with severe aortic stenosis who underwent TAVR.^41^

However, while studies have shown the relationship between the NLR and LMR as components of the NPS and SVG patency,^18,20^ there is a lack of direct evidence linking the NPS with SVG disease in the literature. In our study, we observed a significant increase in SVG disease among patients with high NPS values. This finding could be attributed to the similarities between the parameters of the NPS and the causes of SVG occlusion, such as inflammation and malnutrition. Therefore, the NPS may prove useful in assessing and predicting SVG patency in patients undergoing CABG.

This study has several limitations that need to be acknowledged. First, its retrospective design limits the assessment of the NPS, as only admission NPS values were evaluated with no follow-up assessments. Second, the generalizability of our findings is restricted due to the single-center nature of the study, warranting validation through multicenter studies. Third, our sample size was relatively small, emphasizing the need for further validation through prospective studies with larger cohorts to confirm and generalize our results. The inclusion of patients with acute coronary syndrome may affect the validity of the NPS as the inflammatory and metabolic responses in these patients differ from those with stable coronary artery disease, potentially influencing the score’s accuracy.

## Conclusion

The NPS, a valuable tool for assessing inflammation and nutritional status, may offer insights into SVG patency after CABG surgery. Patients with elevated NPS following CABG should undergo careful monitoring for the onset of SVG disease. Furthermore, preoperative assessment of the NPS could aid in determining the optimal duration of dual antiplatelet therapy and advocating for the use of arterial grafts with superior patency rates.
